# Modeling the Switching Behavior of Functional Connectivity Microstates (FCμstates) as a Novel Biomarker for Mild Cognitive Impairment

**DOI:** 10.3389/fnins.2019.00542

**Published:** 2019-06-11

**Authors:** Stavros I. Dimitriadis, María Eugenia López, Fernando Maestu, Ernesto Pereda

**Affiliations:** ^1^Cardiff University Brain Research Imaging Centre, School of Psychology, Cardiff University, Cardiff, United Kingdom; ^2^Neuroinformatics Group, Cardiff University Brain Research Imaging Centre, School of Psychology, Cardiff University, Cardiff, United Kingdom; ^3^Division of Psychological Medicine and Clinical Neurosciences, School of Medicine, Cardiff University, Cardiff, United Kingdom; ^4^School of Psychology, Cardiff University, Cardiff, United Kingdom; ^5^Neuroscience and Mental Health Research Institute, School of Medicine, Cardiff University, Cardiff, United Kingdom; ^6^MRC Centre for Neuropsychiatric Genetics and Genomics, School of Medicine, Cardiff University, Cardiff, United Kingdom; ^7^Department of Experimental Psychology, Cognitive Processes and Speech Therapy, Universidad Complutense de Madrid, Madrid, Spain; ^8^Laboratory of Cognitive and Computational Neuroscience, Center for Biomedical Technology, Universidad Complutense de Madrid – Universidad Politécnica de Madrid, Madrid, Spain; ^9^Networking Research Center on Bioengineering, Biomaterials and Nanomedicine (CIBER-BBN), Madrid, Spain; ^10^Electrical Engineering and Bioengineering Group, Department of Industrial Engineering and Institute of Biomedical Technology, Universidad de La Laguna, Tenerife, Spain

**Keywords:** magnetoencephalography, mild cognitive impairment, dynamic functional connectivity, resting state, brain states, chronnectome analysis, symbolic dynamics, connectomic biomarker

## Abstract

The need for designing and validating novel biomarkers for the detection of mild cognitive impairment (MCI) is evident. MCI patients have a high risk of developing Alzheimer’s disease (AD), and for that reason the introduction of novel and reliable biomarkers is of significant clinical importance. Motivated by recent findings on the rich information of dynamic functional connectivity graphs (DFCGs) about brain (dys) function, we introduced a novel approach of identifying MCI based on magnetoencephalographic (MEG) resting state recordings. The activity of different brain rhythms {δ, 𝜃, α1, α2, β1, β2, γ1, γ2} was first beamformed with linear constrained minimum norm variance in the MEG data to determine 90 anatomical regions of interest (ROIs). A DFCG was then estimated using the imaginary part of phase lag value (iPLV) for both intra-frequency coupling (8) and cross-frequency coupling pairs (28). We analyzed DFCG profiles of neuromagnetic resting state recordings of 18 MCI patients and 22 healthy controls. We followed our model of identifying the dominant intrinsic coupling mode (DICM) across MEG sources and temporal segments, which further leads to the construction of an integrated DFCG (iDFCG). We then filtered statistically and topologically every snapshot of the iDFCG with data-driven approaches. An estimation of the normalized Laplacian transformation for every temporal segment of the iDFCG and the related eigenvalues created a 2D map based on the network metric time series of the eigenvalues (NMTS^eigs^). The NMTS^eigs^ preserves the non-stationarity of the fluctuated synchronizability of iDCFG for each subject. Employing the initial set of 20 healthy elders and 20 MCI patients, as training set, we built an overcomplete dictionary set of network microstates (n μstates). Afterward, we tested the whole procedure in an extra blind set of 20 subjects for external validation. We succeeded in gaining a high classification accuracy on the blind dataset (85%), which further supports the proposed Markovian modeling of the evolution of brain states. The adaptation of appropriate neuroinformatic tools that combine advanced signal processing and network neuroscience tools could properly manipulate the non-stationarity of time-resolved FC patterns revealing a robust biomarker for MCI.

## Introduction

The major cause of clinical dementia in the elderly is that of Alzheimer’s type (DAT; Qiu et al., 2009), which is mainly characterized by loss of synapses, the accumulation of the Beta amyloid protein (Aβ) and the phosphorylation of the Tau protein. Due to the progressive loss of synapses, which alters the efficient communication within and between various brain subsystems, the DAT may be considered a disconnection syndrome (Delbeuck et al., 2003). The pathological changes of DAT start decades before the first clinical symptoms appear, thus it is important to design proper analytic pathways for analyzing neuroimaging datasets via, e.g., the notion of brain connectivity, which allows the early detecting of such changes (Gómez et al., 2009a; Stam et al., 2009; Maestú et al., 2015). It is extremely important to Alzheimer’s disease (AD) research to identify early on preclinical and prodromal AD as it can assist clinical trials and targeted interventions (Livingston et al., 2017).

Mild cognitive impairment (MCI) is considered to be an intermediate clinical stage between the normal cognitive decline and DAT (Petersen and Negash, 2008). The main parts of the affected brain during the MCI, apart from those involved in action and thought, are those related to memory. For that reason, MCI patients face memory problems on a higher level compared to normal aged population but with no prevalent characteristic symptomatology of dementia-like reasoning or impaired judgment (Petersen et al., 2009). MCI is a heterogeneous state with different subtypes, which complicates in many cases the prediction of DAT (Portet et al., 2006). Additionally, it is also difficult to accurately discriminate symptomatic predementia (MCI) from healthy aging or dementia (DAT) (Petersen and Negash, 2008). Despite these difficulties to achieve an early diagnosis, an accurate identification of MCI should be attempted. Early diagnosis of MCI, even in the absence of a healing strategy, is significant for both pharmacological and non-pharmacological interventions. For that reason, new tools based on neuroimaging approaches are needed to increase sensitivity in the detection of MCI.

Analysis of magnetoencephalographic (MEG) recordings untangled the association between neural oscillations, functional connectivity assessment and neurophysiological activity (Brookes et al., 2011). Altered frequency-dependent functional patterns have been linked to the progression of cognitive decline (Poza et al., 2007). Alternative scenarios of analyzing MEG recordings include single channel analysis, e.g., power analysis, functional connectivity, and brain network analysis in resting state and also in task-based experiments (for a review, see Mandal et al., 2018). Analysis of single channel recordings is a less complex approach that identified aberrant oscillations in AD primarily in the left temporal-parietal-occipital brain areas (Gómez et al., 2009b). Functional connectivity (FC) and effective connectivity (EC) analysis revealed a loss of connectivity in AD compared to healthy control (HC) subjects found mostly in higher frequency bands (Gómez et al., 2017) while multiplex network analysis of MEG study in AD identified affected regions of the hippocampus, posterior default mode network (DMN) and occipital areas (Yu et al., 2017). However, the current clinical literature is limited and no strong conclusion can be drawn.

A recent multicenter MEG study addressed this issue using FC analysis (Maestú et al., 2015). It revealed hypersynchronization in MCI as the most discriminative feature of brain connectivity, mainly over the fronto-parietal and inter-hemispheric links. This pattern was stable across the five different neuroimaging centers that participated in the study (Accuracy ∼ = 80%), which might thereby be considered as a preclinical connectomic biomarker for MCI/DAT. Previous MEG studies based on connectivity analysis described a less organized functional brain network, a hypersynchrony in the fronto-parietal network in MCI subjects (Bajo et al., 2010; Buldú et al., 2011), while patients with DAT demonstrated a less synchronized brain network accompanied with cognitive decline (Stam et al., 2009). This hypersynchronization might be a compensatory mechanism but it cannot be adaptive since the patient’s network is closer to a random network compared to healthy elderly controls (Buldú et al., 2011). In a recent MEG study comparing progressive MCI and stable MCI, authors described hypersynchronization in the α band between the anterior cingulate and posterior brain areas in the progressive MCI group (López et al., 2014).

Spontaneous fluctuations of functional MRI (fMRI) blood-oxygen-level-dependent (BOLD) signals are temporally coherent between distinct spatial brain areas and not random. Biswal et al. (1995) demonstrated that fluctuations from motor areas were correlated even in the absence of a motor task. FC based on BOLD signal is modulated by cognitive and affective states (Richiardi et al., 2011; Shirer et al., 2012), by learning (Bassett et al., 2011), and also spontaneously (Kitzbichler et al., 2009; Britz et al., 2010; Chang and Glover, 2010).

When non-stationarity is taken into account and a dynamic functional connectivity (DFC) approach is adopted for studying FC patterns even in the absence of a task (resting state), more sophisticated algorithmic analyses should be used. In this line, two studies have recently been published simultaneously that presented a data-driven methodology. In the first one, Allen et al. (2014) proposed a method based on *k*-means clustering, aimed at detecting distinct “FC states” in the resting brain. These authors clearly showed differences from the stationary static functional brain networks. The second study proposed a data-driven method focused on extracting, out of hundreds of functional connectivity graphs (FCGs) in a multi-trial experimental paradigm, distinct brain states called *functional connectivity microstates* (FCμstates; Dimitriadis et al., 2013a). Both approaches revealed the need of dynamic FC to explore brain dynamics via the notion of brain connectivity, as it is clear that brain FC “hops” from one state to another (FCμstate) leading to a Markovian chain with characteristic favored transitions between distinct pairs of FCμstates (Dimitriadis et al., 2010b, 2013a,b; Allen et al., 2014).

In the last years, an increasing amount of human brain research based on functional imaging methods (electro-encephalography: EEG/magnetoencephalography: MEG/functional Magnetic Resonance Imaging: fMRI) has adopted a dynamic approach for exploring how brain connectivity fluctuates during resting-state and tasks alike (Laufs et al., 2003; Mantini et al., 2007; Dimitriadis et al., 2009, 2010b, 2012b, 2013a,b, 2015a,b,c,d, 2016a,b; Chang and Glover, 2010; Bassett et al., 2011; Handwerker et al., 2012; Ioannides et al., 2012; Hutchison et al., 2013; Liu and Duyn, 2013; Allen et al., 2014; Braun et al., 2014; Mylonas et al., 2015; Toppi et al., 2015; Yang and Lin, 2015; Calhoun and Adali, 2016). The aforementioned studies have demonstrated the superiority of DFC as compared to a static connectivity analysis.

In parallel, the concept of cross-frequency coupling (CFC) is gaining attention lately in the neuroscience community, as evinced by the increasing number of papers published with the incorporation of this type of interaction in the analysis (van Wijk and Fitzgerald, 2014; Dimitriadis et al., 2015a,c, 2016a,b; Florin and Baillet, 2015; Antonakakis et al., 2016a,b; Tewarie et al., 2016). Specifically, intrinsic coupling modes and especially CFC bias the task-related response and are sensitive to various brain diseases and disorders such as DAT, Parkinson, etc. (see, e.g., Engel et al., 2013 for a review). More recent studies have shown that the dynamics of spontaneously generated neural activity can be informative regarding the functional organization of large-scale brain networks (Fox et al., 2005; He et al., 2008; Hipp et al., 2012), revealing intrinsically generated “coupling modes” at multiple spatial and temporal scales (Deco and Corbetta, 2011; Engel et al., 2013).

Based on the aforementioned methodological evidence in microscale, it is significant to explore the repertoire of intra- and cross frequency interactions across brain rhythms and brain areas under the same integrated graph model (Dimitriadis et al., 2016a, 2017b, 2018a; Antonakakis et al., 2017).

In a previous study, we demonstrated how to design a connectomic biomarker for MCI based on source-reconstructed MEG activity via static brain network analysis (Dimitriadis et al., 2018a). Here, we extended this work by proposing a scheme to design a dynamic connectomic biomarker under the framework of DFC analysis. Additionally, the proposed scheme will be validated in a second blind dataset (SID did not know anything about the labels).

To this aim, we analyzed the MEG activity of healthy controls and MCI patients at resting-state (eyes-open) via DFC analysis. Based on a previous approach (Dimitriadis et al., 2016a, 2017b), we detected the dominant type of interaction per pair of MEG sources and temporal segment (Dimitriadis, 2018). This approach produced a subject-specific dynamic functional connectivity graph (DFCG). This approach created a 2D matrix of size sources × temporal segments that described the evolution of the eigenvalues across experimental time. Afterward, we used neural gas to design overcomplete dictionaries for sparse representation of NMTS^eigen^ independently for the two groups (Dimitriadis et al., 2013a). Then, we validated the whole approach in a blind dataset to quantify the generalization of the proposed method.

In the Section “Materials and Methods,” we described the data acquisition, preprocessing steps, information about the datasets and the proposed methodological scheme. The Section “Results” is devoted to describing the results—including the prototypical network FCμstates, the accuracy of prediction in a blind dataset and network-based information of brain states. Finally, the Section “Discussion” includes the discussion of the current research results with future extensions.

## Materials and Methods

### Subjects and Ethics Statement

The training dataset includes 18 right-handed individuals with MCI (71.89 ± 4.51 years of age), and 22 age- and gender-matched neurologically intact controls (70.91 ± 3.85 years of age) were also recorded. Table 1 summarizes their demographic characteristics. All participants were recruited from the Neurological Unit of the “The Hospital Universitario San Carlos,” Madrid, Spain. They were right-handed (Oldfield, 1971) and native Spanish speakers. We used also a set of 20 subjects of unknown label (blind author SD) for further validation of the proposed dynamic connectomic biomarker (DCB). Table 2 summarizes the mean and standard deviation of the demographic characteristics of controls and MCI subjects from the blind dataset. Including the blind subjects, the total sample consisted of 29 MCI and 31 controls. At the beginning, we used 18/22 subjects for MCI/control group, correspondingly to train the algorithm and we kept 20 (nine control subjects and 11 MCI) for blind classification.

**Table 1:** Mean ± standard deviation of the demographic characteristics of controls and MCIs.

	Age	Gender (M/F)	Educational level	MMSE	LH ICV	RH ICV
Control (*n* = 22)	70.91 ± 3.853	9/13	3.50 ± 1.225	29.32 ± 0.646	0.0025 ± 0.0003	0.0025 ± 0.0003
MCI (*n* = 18)	71.89 ± 4.510	7/11	2.71 ± 1.359	27.24 ± 1.954	0.0022 ± 0.0005	0.0021 ± 0.0005

*M = males; F = females; Educational level was grouped into five levels: (1) Illiterate, (2) Primary studies, (3) Elementary studies, (4) High school studies, and (5) University studies; MMSE = Mini-Mental State Examination; LH ICV, Left hippocampus normalized by total intracranial volume (ICV); RH ICV, Right hippocampus normalized by ICV.*

**Table 2:** Mean ± standard deviation of the demographic characteristics of the blind sample of controls and MCIs.

	Age	Gender (M/F)	Educational level	MMSE	LH ICV	RH ICV
Control (*n* = 9)	70.22 ± 3.8333	1/8	3.44 ± 1.333	29.33 ± 0.707	0.0026 ± 0.0005	0.0027 ± 0.0004
MCI (*n* = 11)	73.45 ± 3.297	7/4	3.91 ± 1.221	26.90 ± 2.132	0.0018 ± 0.0004	0.0021 ± 0.0004

*M = males; F = females; Educational level was grouped into five levels: (1) Illiterate, (2) Primary studies, (3) Elementary studies, (4) High school studies, and (5) University studies; MMSE = Mini-Mental State Examination; LH ICV, Left hippocampus normalized by total intracranial volume (ICV); RH ICV, Right hippocampus normalized by ICV.*

To explore their cognitive and functional status, all participants were screened by means of a variety of standardized diagnostic instruments and underwent an extensive cognitive assessment, as described in López et al. (2016).

Mild cognitive impairment diagnosis was established according to the National Institute on Aging-Alzheimer Association (NIA-AA) criteria (Albert et al., 2011), with all of them being categorized as “MCI due to AD intermediate likelihood.” They met the clinical criteria and also presented hippocampal atrophy, which was measured by magnetic resonance (MRI). According to their cognitive profile, they were also classified as amnestic subtype (Petersen et al., 2001).

The whole sample was free of significant medical, neurological and/or psychiatric diseases (other than MCI). Exclusion criteria included: a modified Hachinski Ischemic score ≥4 (Rosen et al., 1980); a geriatric depression scale short-form score ≥5 (Yesavage et al., 1983); a T2-weighted MRI within 12 months before MEG screening with indication of infection, infarction or focal lesions (rated by two independent experienced radiologists, Bai et al., 2012); and other possible causes of cognitive decline such as B_12_ deficit, diabetes mellitus, thyroid problems, syphilis or human immunodeficiency virus (HIV). Finally, those participants with medical treatment that could affect MEG activity (e.g., cholinesterase inhibitors) were required to interrupt it 48 h before the MEG recordings.

The present study was approved by the local ethics committee and all subjects signed an informed consent prior to their MEG recording.

### MRI Acquisition and Hippocampal Volumes

Three-dimensional T1-weighted anatomical brain magnetic MRI scans were collected with a General Electric 1.5 TMRI scanner, using a high resolution antenna and a homogenization PURE filter (Fast Spoiled Gradient Echo (FSPGR) sequence with parameters: TR/TE/TI = 11.2/4.2/450 ms; flip angle 12°; 1 mm slice thickness, a 256 × 256 matrix and FOV 25 cm). Freesurfer software (version 5.1.0.; Fischl et al., 2002) was used to obtain the hippocampal volumes, which were normalized with the overall intracranial volume (ICV) of each subject.

### MEG Acquisition and Preprocessing

4 min of eyes-open resting state data were recorded while the participants were seated in a 306-channel (one magnetometer and two orthogonal planar gradiometers per recording site, sampling frequency of 1 kHz) Vectorview system (Elekta Neuromag) placed in a magnetically shielded room (VacuumSchmelze GmbH, Hanau, Germany) at the “Laboratory of Cognitive and Computational Neuroscience” (Madrid, Spain). Subjects had to fix their gaze at a cross, which was projected in a screen. The position of the head relative to the sensor array was monitored by four head position indicator (HPI) coils attached to the scalp (two on the mastoids and two on the forehead). These four coils along with the head shape of each subject (referenced to three anatomical fiducials: nasion and left-right preauricular points) were acquired by using a three-dimensional Fastrak Polhemus system. Vertical ocular movements were monitored by two bipolar electrodes, which were placed above and below the left eye, and a third one on the earlobe, for electrical grounding.

Four HPI coils were placed in the head of the subject, two in the forehead and two in the mastoids, for an online estimate of the head position. The HPI coils were fed during the whole acquisition, allowing for offline estimation of the head position.

Maxfilter software (version 2.2 Elekta Neuromag) was used to remove the external noise from the MEG data using the temporal extension of signal space separation (tSSS) with movement compensation (correlation threshold = 0.9 m time window = 10 s) (Taulu and Simola, 2006). This algorithm removes the signals, whose origin is estimated outside the MEG helmet, while keeping intact the signals coming from inside the head. In addition, the continuous HPI acquisition, combined with the tSSS algorithm, allowed continuous movement compensation. As a result, the signal used in the next steps came from a set of virtual sensors whose position remained static in respect to the head of the subject. Recordings from those subjects whose movement along the recording was larger than 25 mm were discarded, following the recommendations of the manufacturer.

### Source Reconstruction

We generated a volumetric grid for the MNI template by adopting a homogenous separation of 1 cm in each direction, with one source placed in (0, 0, 0) in MNI coordinates. The whole procedure resulted in a source model with 2,459 sources inside the brain surface where each one consisted of three perpendicular dipoles. Every source was then labeled using the automated anatomical labeling (AAL) atlas (Tzourio-Mazoyer et al., 2002). We finally considered 1,467 cortical sources. The computed grid was then transformed to subject specific space employing the original T1 image. The realignment of the grid and brain surface was realized manually to the Neuromag coordinate system following the three fiducials and the head shape guides. Employing a realistically shaped head, we estimated a lead field (Nolte, 2003). We source reconstructed frequency-dependent brain activity using a Linearly Constrained Minimum Variance (LCMV) beamformer (Van Veen et al., 1997). We ran the LCMV beamformer independently for the following eight frequency bands: δ (1–4 Hz), 𝜃 (4–8 Hz), α_1_ (8–10 Hz), α_2_ (10–13 Hz), β_1_ (13–20 Hz), β_2_ (20–30 Hz), γ_1_ (30–49 Hz), and γ_2_ (51–90 Hz). The resulting spatial filters were projected over the maximal radial direction, getting only one spatial filter per source. “Radial direction” means the direction of the segment connecting the dipole location to the center of the sphere best approximating the brain surface. Radial dipoles in a spherical conductor do not produce a magnetic field outside of the conductor (Sarvas, 1987), so this projection avoids the creation of undetectable sources among the target dipoles. Finally, we represented every brain area region of interest according to the AAL atlas by one source-space time series per frequency band using two alternative solutions: (1) the PCA of all the sources in the area or (2) the source closest to the centroid of the area (CENT).

**FIGURE 1:**
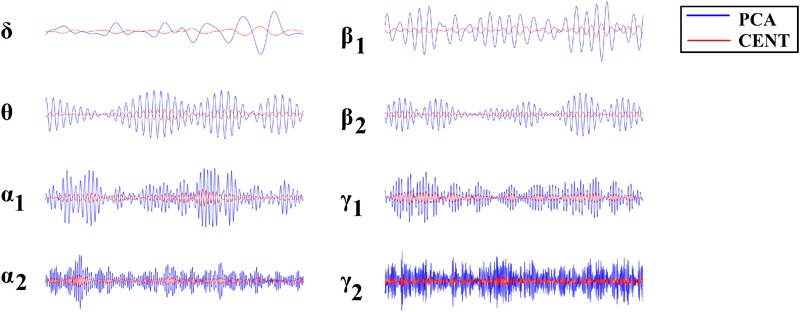
ROI Virtual Sensor representation of left precentral gyrus magnetoencephalographic activity from the first healthy control subject. Virtual sensor time series with blue and red color represent brain activity for **(A)** PCA and **(B)** CENT time series, respectively.

Figure 1 illustrates the source-localization procedure and the different frequency-dependent representative Virtual Sensor time series for the two ROI representation schemes, PCA and the CENT.

### Dynamic Functional Connectivity Graphs (DFCGs)

#### Construction of the Integrated DFCGs

The DCFG analysis was restricted to the 90 ROIs of the AAL atlas. Adopting a common sliding window of width equal to 1 s to get at least 1 cycle of δ activity and a moving step of 50 ms, we estimated the dynamic networks for both intra-frequency (8 frequency bands) and inter-frequency coupling modes (8^∗^7/2 = 28 cross-frequency pairs) using the following formula of the imaginary part of phase locking value (iPLV).

(1) $$iPLV={\frac{1}{T}}^{*}\left|Im\left(\sum_{t=1}^{T}e^{i(\varphi_{i}(t)-\varphi_{j}(t))}\right)\right|,$$

where *ϕ(t)* is the phase of the signal in the corresponding frequency band (intra-frequency modes) and between frequencies (CFCs). For further details regarding phase-to-amplitudeCFC, see Dimitriadis et al. (2015a) and the Section “Construction of the Integrated Dynamic Functional Connectivity Graph” in Supplementary Material.

This procedure, whose implementation details can be found elsewhere (Dimitriadis et al., 2010b, 2015a, 2016a, 2017a,b, 2018b), resulted in a four-dimensional tensor of size [coupling modes × temporal segments × ROIs × ROIs] or [36 × 2,401 × 90 × 90] time-varying PAC graphs per participant (^TV^PAC). Following proper surrogate analysis and a framework which have been presented in a previous study (Dimitriadis et al., 2018b), we defined the dominant intrinsic coupling mode (DICM) per pair of sources and across temporal segments. This procedure generates two three-dimensional tensors of size [temporal segments × ROIs × ROIs]. The first one keeps the functional coupling strength (iPLV) across anatomical space and time, while the second tabulates the DICM using an index for every possible case : {1 for δ, 2 for 𝜃, 3 for α_1_, …,8 for γ_2_, 9 for δ-𝜃,..., 36 for γ_1_-γ_2_}. The following section describes briefly the surrogate analysis appropriate for reducing pitfalls in CFC analysis and also to define the DICM.

#### Statistical Filtering Scheme

First, we must identify true CFC interactions that are not driven by the changes in signal power. Secondly, following a proper surrogate analysis our DICM model can detect the DICM between every pair of sources and at every temporal segment. The whole procedure of analysis is described elsewhere in detail in Dimitriadis et al. (2016a), Dimitriadis and Salis (2017), and Dimitriadis (2018) and also in the Section “From Prominent Intrinsic Coupling Modes to Dominant Intrinsic Coupling Modes” in Supplementary Material.

**FIGURE 2:**
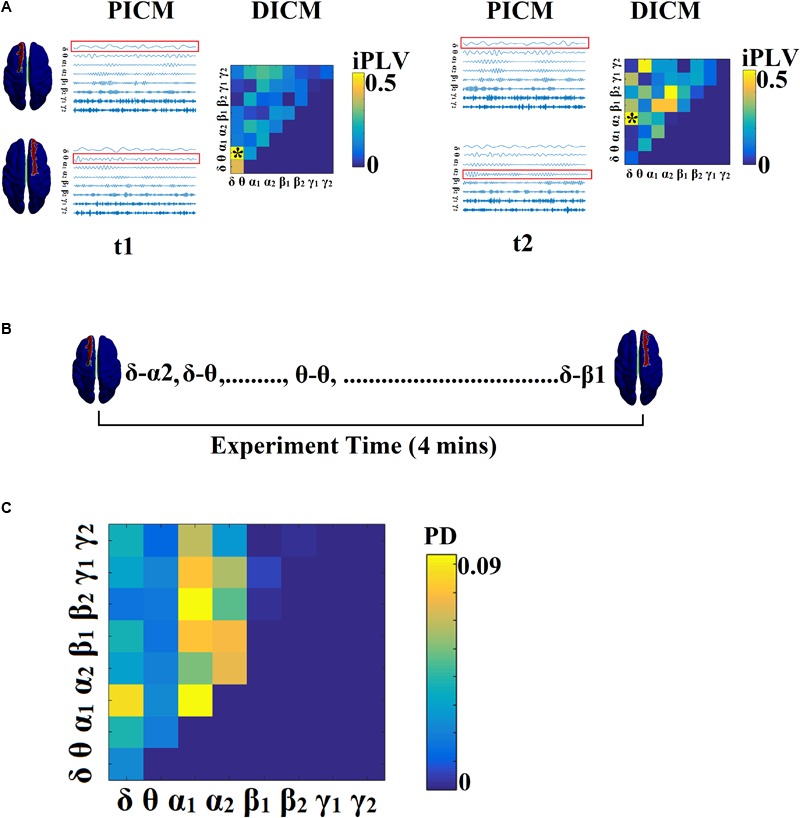
Determining DICM. An example for AU task derived from the first trial of the first subject. **(A)** Schematic illustration of our approach employed to identify the DICM between two sources (Left superior frontal gyrus, Right superior frontal gyrus) for two consecutive sliding time windows (t_1_, t_2_) during the first 4 s of resting-state activity from the first healthy control subject. In this example, the functional synchronization between band-passed signals from the two sources was estimated by imaginary Phase Locking (iPLV). In this manner iPLV was computed between the two sources either for same-frequency oscillations (e.g., δ to δ…, γ_2-_γ_2_; 8 intra-frequency couplings) or between different frequencies (e.g., δ to 𝜃, δ to α_1_..., γ_1-_γ_2_; 28 cross-frequency pairs). The sum of 8 + 28 = 36 refers to Potential Intrinsic Coupling Modes (PICM), which are tabulated in a matrix format. In the main diagonal, we inserted the intra-frequency couplings while in the off-diagonal the cross-frequency pairs were inserted. Statistical filtering, using surrogate data for reference, was employed to assess whether each iPLV value was significantly different from chance. During t_1_ the DICM reflected significant phase locking between α_1_ and α_2_ oscillations (indicated by red rectangles) in the oscillation list and a “^∗^” in the comodulogram. The DICM remains stable also for the t_2_ between α_1_ and α_2_ oscillations whereas during t_3_ the dominant interaction was detected between 𝜃 and α_2_ oscillations. **(B)** Burst of DICM between Left and Right superior frontal gyrus. This packing can be thought to associate the “letters” contained in the DICM series to form a neural “word,” a possible integration of many DICMs. From this burst of DICM, we can quantify the probability distribution (PD) of DICM across experimental time (see **C**). **(C)** Tabular representation of the probability distribution (PD) of DICM for left and right superior frontal gyrus across the experimental time shown in **B**. This matrix is called a comodulogram and keeps the information of PD from the 36 possible coupling modes. In the main diagonal the PD of the 8 possible intra-frequency coupling can be seen while in the off-diagonal are the 28 possible cross-frequency pairs. PICM, Prominent Intrinsic Coupling Modes; DICM, Dominant Intrinsic Coupling Modes; iPLV, imaginary part of Phase Locking Value; PD, probability distribution.

Figure 2 illustrates the whole procedure of the DICM model for the first two temporal segments of resting-state activity of the first healthy control subject.

**FIGURE 3:**
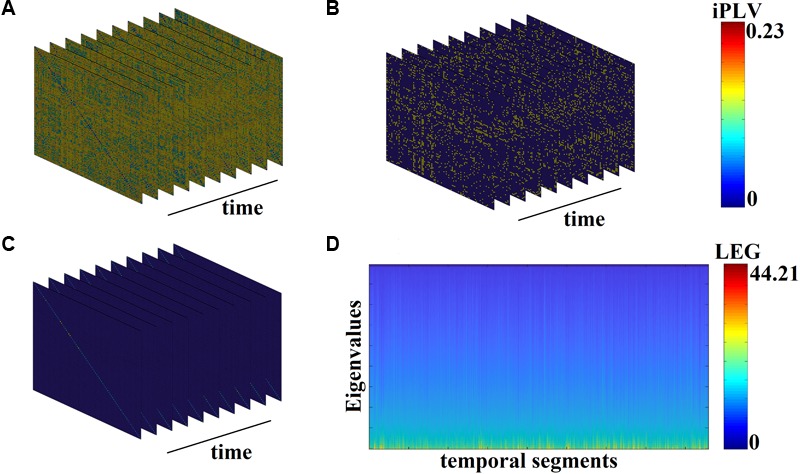
From DFCG to the temporal evolution of Laplacian eigenvalues (LEG; from the first healthy control subject). **(A)** The first 10 snapshots of DFCG. **(B)** The quasi-static FCGs shown in A were topologically filtered with OMST. **(C)** The normalized Laplacian transformation of the topologically filtered FCGs shown in B. **(D)** Temporal evolution of the Laplacian eigenvalues for the 2,401 temporal segments.

Figure 3A demonstrates the first 10 snapshots of the DFCG from the first healthy control subject.

#### Topological Filtering Scheme Based on OMSTs

Apart from surrogate analysis, which is a statistical filtering procedure of the functional couplings within an FCG akin to a regularization to sparsify the 4D array described above, we adopted a topological filtering to further enhance the network topology and the most significant interactions. To this aim, we applied a novel data-driven thresholding scheme, proposed by our group and termed Orthogonal Minimal Spanning Trees (OMSTs; Dimitriadis et al., 2017a,b), to each FCG derived from each subject and temporal segment independently.

Figure 3B demonstrates the temporal evolution of the topologically filtered dFCG for the first 10 temporal segments.

#### Graph Signal Processing

After extracting the most significant connections in DCFGs from each individual, we transformed every snapshot of the DFCG into the graph Laplacian variant called the normalized Laplacian matrix. With A being the functional connectivity graph and D being the degree matrix containing the degree of every node in the main diagonal, graph Laplacian L can be defined as L = D – A. The normalized graph Laplacian is defined as L_sym_ = D^-1/2^LD^-1/2^ (Shuman et al., 2013). We estimated the sorted eigenvalues of the Lsym for every snapshot of DFCG resulting in a two-dimensional matrix of size [source (90) × temporal segments (2.401)] per subject. These two-dimensional matrices were concatenated separately for the healthy control and disease group of the training set. Practically, the concatenation was performance was performed along the temporal direction.

Figure 3C shows the temporal evolution of the normalized Laplacian transformation of the dFCG for the first 10 temporal segments while Figure 3D is dedicated to the temporal evolution of the eigenvalues.

#### A Vector-Quantization (VQ) Modeling of Group NMTS^eigen^

This subsection describes briefly our symbolization scheme, presented in greater details elsewhere (Dimitriadis et al., 2011, 2012a, 2013a,b). The group-specific **NMTS^eigen^** patterns can be modeled as prototypical FC microstates (FCμstates). In our previous studies, we demonstrated a better modeling of DFCG based on vector quantization approach (Dimitriadis et al., 2013a, 2017b, 2018b). A codebook of *k* prototypical FC states (i.e., functional connectivity microstates-FCμstates) was first designed by applying the neural-gas algorithm (Dimitriadis et al., 2013a). This algorithm is an artificial neural network model, which converges efficiently to a small number *k* of codebook vectors, using a stochastic gradient descent procedure with a soft-max adaptation rule that minimizes the average distortion error (Martinetz et al., 1993). A neural-gas algorithm has been applied independently to each group by concatenating the 2D matrix of size [2.401 × 90] that describes the fluctuation of Laplacian eigenvalues.

The outcome of the neural-gas algorithm over NMTS^eigen^ is the construction of a symbolic sequence of group-specific prototypical FCμstates, one per subject. An example of such a symbolic time series (STS) is a Markovian chain with three FCμstates: {1, 2, 3, 2, 1, 3, 2…} where each integer defines a unique brain state (FCμstates) assigned to every quasi-static temporal segment.

#### External Validation in a Blind Dataset

We designed a novel approach for classifying a blind subject. We reconstructed the subject-specific NMTS^eigen^ with both HC-based prototypical FCμstates and MCI-based prototypical FCμstates. Specifically, for every temporal segment expressed via a vector of 90 eigenvalues we estimated which of the prototypical FCμstates is much closer, employing Euclidean distance for an appropriate criterion. Under this scheme, we rebuilt the original NMTS^eigen^ twice, once using prototypical FCμstates of HC and once using prototypical FCμstates of MCI. Then, we estimated the reconstruction mean squared error between the original NMTS^eigen^ and the two rebuilt NMTS^eigen^ based on prototypical FCμstates. Finally, we assigned the test sample to the class with the lowest reconstruction error (see Figure 6).

### Markov Chain Modeling for Synchro State Transitions

The temporal sequence of spontaneous activity can be modeled as a Markovian process, which predicts the probabilities of several discrete states recurring or switching among themselves at different time points analyzing time-point-based brain activity (Van de Ville et al., 2010; Gärtner et al., 2015). Several studies have investigated transition probabilities between phase-synchronized states on a sub-second temporal scale, untangling the Markovian property and the switching behavior of finite network-level brain states (Dimitriadis et al., 2013c, 2015b; Baker et al., 2014; Jamal et al., 2014).

#### Markovian Process of Time-Sequential FCμstates

A Markov model describes the underlying dynamical nature of a system that follows a chain of linked states, where the appearance of a state at any given instant depends only on the preceding ones (Gagniuc, 2017). In the Markov chain modeling for synchrostate transitions during the deductive reasoning and task-free processes, the first order transition matrices were estimated in a probabilistic framework. According to discrete-time Markov chain theory (Jarvis and Shier, 1999), a finite number (S_1_, S_2_…, S_m_) of inferred states that evolve in discrete time with a time-homogeneous transition structure can be mathematically represented by either its transition probability matrix or its directed graph (digraph). Here, the inferred states refer to the prototypical FCμstates. A feasible transition is one whose occurring probability is greater than zero. The probability of transition from node (state) *i* to node *j* is defined as

(2) $$P_{ij}\text{=}\frac{N_{ij}}{\sum_{ij}N_{ij}},i\text{=}1,2,\ldots ,nj\text{=}1,2,\ldots ,m,$$

where *Nij* is the number of transitions from node *i* to node *j*. Obviously, the sum of the transition probabilities along each row of the transition matrix *P* equals one. The complete digraph for a finite-state Markov process has edges of transition probabilities between every node *i* and every other node *j*. Here, nodes refer to FCμstates in the Markov chain. In the digraphs created in this study, *P_ij_* survives a *p*-value derived from 10,000 shuffled-surrogates of the original STS.

#### Temporal Measurements of an FCμstate Symbolic Sequence

For further summarizing inter-FCμstate transition patterns, relevant temporal measurements were obtained and analyzed from the Markov chain structures of the subject-specific FCμstate sequence, including: (1) fractional occupancy for each class of FCμstate (i.e., the fraction of the number of distinct FCμstate of a given class occurring within 2,401 temporal segments), (2) dwell time for each FCμstate which gives the average time the brain spends within a specific FCμstate in consecutive temporal segments, (3) transition probabilities (TP) of a given FCμstate to any other functional connectivity state, (4) the complexity index (CI) that quantifies the richness of the spectrum of code words formed up to a length based on the symbolic time series (Dimitriadis, 2018), and (5) the flexibility index (FI) that quantifies the transition of the brain states (FCμstates) between consecutive temporal segments.

#### Assessing the Statistically Significant Level of the Symbolic-Based Estimates

To assess the statistically significant level of the aforementioned four estimates (excluding CI), we shuffled the group symbolic time series 10,000 times and re-estimated the surrogate-based *p*-values for every estimate per subject. CI is normalized by default with surrogates.

### Linking MMSE With Chronnectomics

To investigate the possible relation between MMSE and the chronnectomics derived by the FCμstate symbolic sequence (see section “Temporal Measurements of an FCμstate Symbolic Sequence”), we used the canonical correlation analysis (CCA) approach to see whether MMSE correlates with seven chronnectomic variables. In our analyses, the significance of the correlation was estimated using Bartlett’s approximate chi-squared statistic as implemented in MATLAB.

### Algorithms and MATLAB Code

All the algorithmic steps of constructing the DCFGs were implemented on inhouse software written in MATLAB, freely available from the first author’s website. LCMV beamformer was programmed under Fieldtrip’s environment (Oostenveld et al., 2011).

## Results

### Group Prototypical FCμstates

**FIGURE 4:**
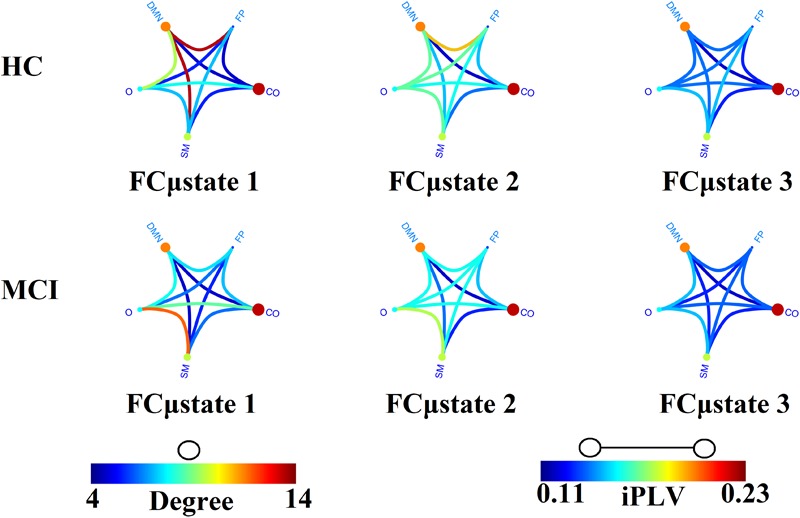
Prototypical FCμstates for healthy control (HC) and MCI. The neural gas algorithm revealed three prototypical FCμstates per group with different spatial patterns. FP, Fronto-Parietal; DMN, Default Mode Network; CO, Cingulo-Opercular; S, Sensorimotor; O, Occipital.

Figure 4 illustrates the prototypical group-specific FCμstates for each group by assigning the 90 AAL brain areas to five well-known brain networks. The size and color of every circle decode the mean degree within every brain network while the color of each connection defines the mean functional strength between every pair of brain networks. FCμstates can be described based on the most connected brain networks focusing on their degree. The most connected brain networks are the DMN and CO. Following a statistical test by comparing the functional coupling strength between FPN and DMN independently for every FCμstate, we found significant higher values for FCμstates 1 and 3 for HC compared to MCI (*p* = 0.00045 for FCμstate 1 and *p* = 0.000012 for FCμstates 3, Wilcoxon Rank Sum Test).

**FIGURE 5:**
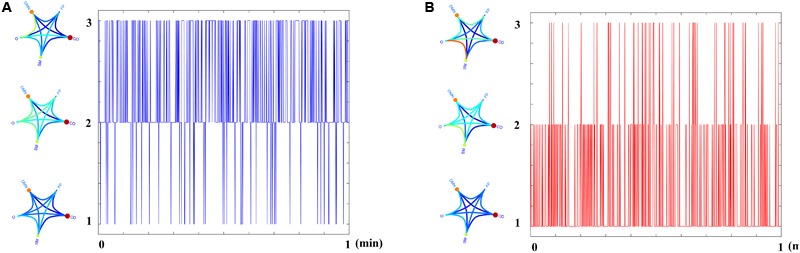
Temporal Evolution of Prototypical FCμstates for the first subject of **(A)** HC and **(B)** MCI group for the 1st min of resting state.

Figure 5 demonstrates the dynamic reconfiguration of prototypical FCμstates for the first subject of both groups for the 1st min.

### Classification of Blind Samples via Representations With Prototypical Netμstates^eigen^

**FIGURE 6:**
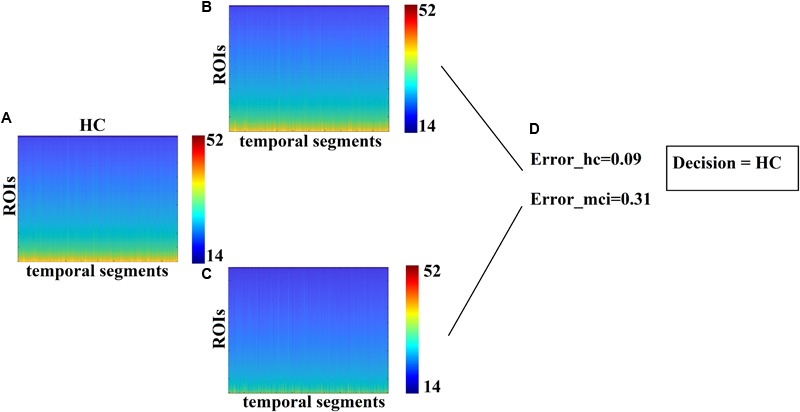
Classification of blind subjects via prototypical Net μstates^eigen^. **(A)** Evolution of eigenvalues for the test sample. **(B,C)** Reconstruction of the temporal evolution of eigenvalues of the train sample with both group-specific prototypical Net μstates^eigen^. **(D)** Estimation of the reconstruction error between the original temporal evolution of eigenvalues in **A**. and the two prototypical-based shown in **B**. The decision of the subject’s label was taken via the lower reconstruction error.

Each test sample with an unknown label was classified to one of the two classes using as a criterion the minimization of the reconstruction error. The minimum reconstruction error denotes the class label of the sample. In our study, we used 20 samples with a distribution of 11 MCIs and 9 controls with 85% accuracy for CENT (17 out of 20) and 70% for the PCA representation scheme (14 out of 20). SID received the blind dataset from MEL, who evaluated the outcome of this research. Figure 6 illustrates the methodological approach. Figure 6A refers to the temporal resolution of the Laplacian eigenvalues of a blind HC subject while Figure 6B,C the reconstruction of Figure 6A matrix employing the Prototypical Net μstates^eigen^ related to HC and MCI, correspondingly. Based on the reconstruction error between the original matrix (Figure 6A) and the two reconstructed matrixes (Figure 6B,C), a decision regarding the label of the blind subject was taken based on the lowest reconstruction error (Figure 6D).

### Group-Differences of Temporal Measurements Derived From FCμstate Symbolic Sequence

**FIGURE 7:**
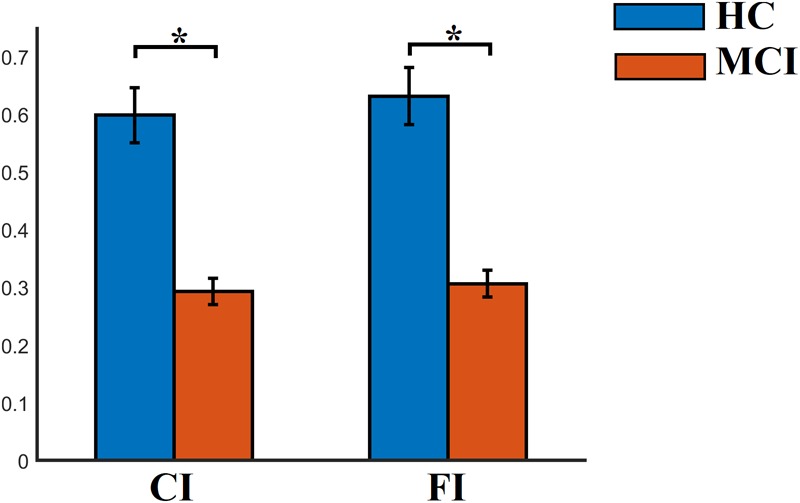
Group-averaged CI and FI. ^∗^Wilcoxon Rank-Sum Test; *p*-value < 0.00000001.

**FIGURE 8:**
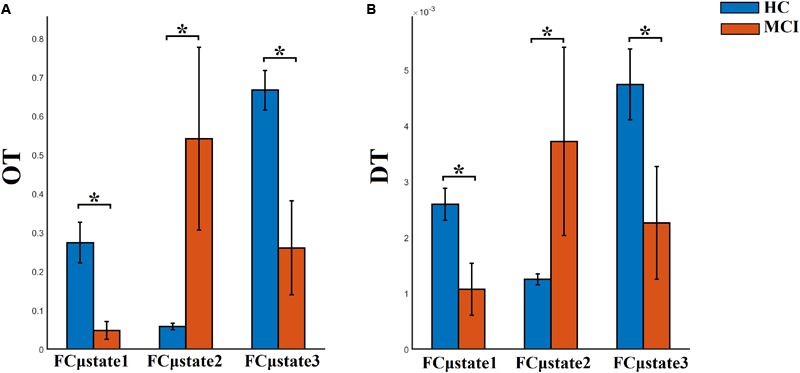
Group-averaged **(A)** OT and **(B)** DT per FCμstate. ^∗^Wilcoxon Rank-Sum Test; *p*-value < 0.00000001.

FI, OT, and DT were significantly higher than the surrogates based values derived from the shuffled symbolic time series (*p* < 0.001). We detected significant higher FI and CI for HC compared to MCI applying a Wilcoxon Rank-Sum test (Figure 7, *p*-value < 0.00000001). Summarizing the results from OT and DT, HC subjects spent significantly higher time compared to MCI to first and third FCμstate while MCI spent significantly more time to the second FCμstate Figure 8, *p*-value < 0.00000001).

### Modeling Dynamic Reconfiguration of Functional Connectivity Graphs as a Markovian Chain

**FIGURE 9:**
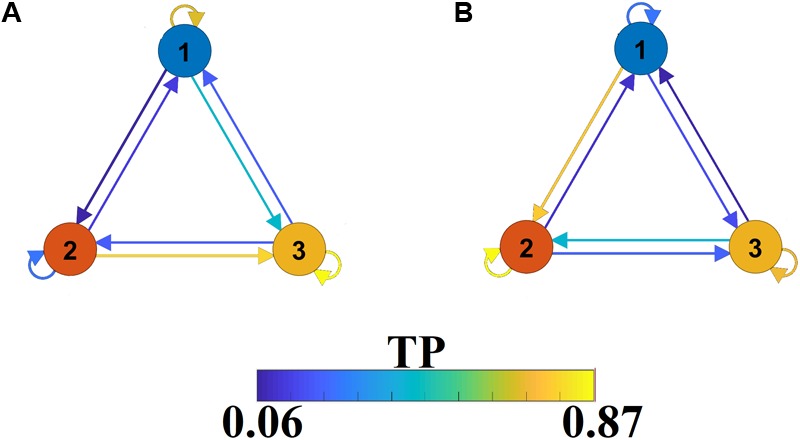
A finite-state diagram showing group-averaged transition probability matrix (TP) of the symbolic time series, which describes the temporal evolution of the brain, states (FCμstate). **(A)** For healthy control and **(B)** forMCI.

The outcome of the VQ modeling of NMTS^eigen^ is the derived Netμstates^eigen^ called FCμstates (see Figure 4), where its evolution is described via a symbolic time series, a Markovian chain. Figure 9 illustrates a well-known scheme of the group-averaged transition probabilities (TP) between the three FCμstates for both groups. Our analysis revealed significant group differences in terms of TP, while the TPs were significantly different compared to the surrogates’ symbolic time series. Self-loops defined the “staying” TP of brain dynamics to the same brain state.

The symbolic time series illustrated in Figure 5 is a Markovian chain of order 1 and it is shown schematically with a diagram of three nodes defining the three FCμstates (Figure 4, 9) while the arrows from one state to the other show the TP. Our results revealed significant group differences between every possible brain state transition (Wilcoxon Rank-Sum test, *p* < 0.0001/9).

### Comodulograms of Dominant Intrinsic Coupling Modes (DICM)

**FIGURE 10:**
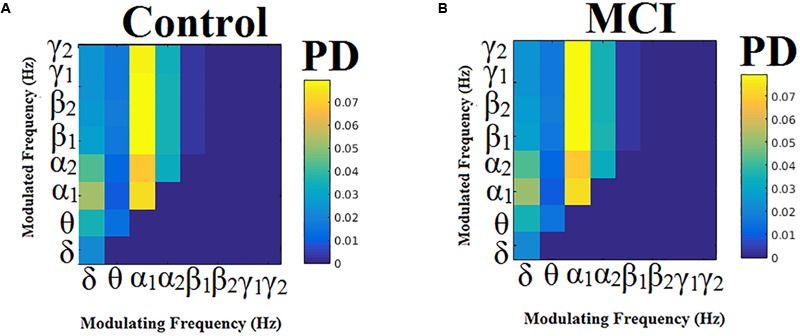
Group-averaged empirical Probability Distribution values of DICMs for MCI **(B)** compared to control group **(A)**.

Probability distributions (PD) of prominent intrinsic coupling modes across all sources pairs and time windows were summarized for each group in the form of an 8 × 8 matrix. The horizontal axis refers to the phase modulating frequency (Hz) while the *y*-axis refers to the amplitude modulated frequency (Hz). The main diagonal of the comodulograms keeps the PD of intra-frequency phase-to-phase coupling. Group-averaged comodulograms in Figure 10 demonstrate the empirical PD of DICM revealing a significant role of α_1_ as phase modulator of the whole studying spectrum up to high-gamma (γ_2_) activity, which covers almost 50% of pairwise source connections and time windows. No significant trend was detected regarding the PD of each pair of frequencies between the two groups (*p* < 0.05, Wilcoxon rank-sum test, Bonferroni corrected). Moreover, no significant difference was found regarding the PD of the groups for every possible pair of sources (*p* < 0.05, Wilcoxon rank-sum test, Bonferroni corrected). Finally, transition dynamics of DICM between consecutive time windows at every source pair did not uncover any group difference (for further details, see Dimitriadis et al., 2016a).

### Correlation of MMSE With Chronnectomics

**FIGURE 11:**
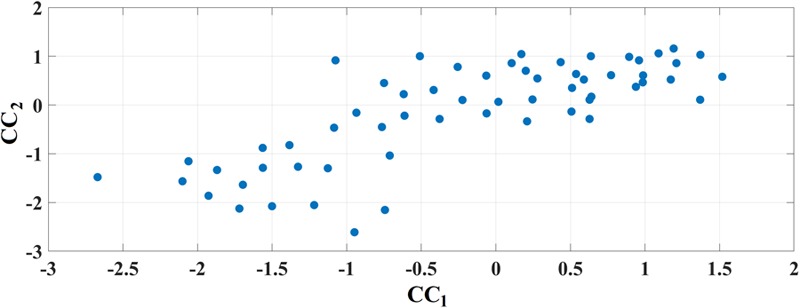
Canonical Correlation Analysis of Chronnectomics and MMSE. Figure plots the canonical variable scores referring to the two sets.

Figure 11 demonstrates the outcome of CCA analysis between chronnectomics and the well-known MMSE. The Chi-square was 26.95 and the related *p*-value = 0.00033886. *x*-axis refers to the canonical variable scores of the chronnectomics, where the DT of the three NMTS^eigen^ contributes most to the maximization of their canonical correlation with MMSE. OC_2 did not associate with the CCA mode of MMSE variability. The 1st canonical component is:

$$CC_{1}\text{=}\;0.11^{*}FI+0.02^{*}OC_{1}+0.02^{*}OC_{3}\pm 0.0056^{*}CI+3.38^{*}DT_{1}+5.76^{*}DT_{2}+2.65^{*}DT_{3}$$

and the second is:

CC2 = 0.59^∗^MMSE.

## Discussion

We have demonstrated here a novel framework for designing a proper DCB for the detection of MCI subjects from spontaneous neuromagnetic activity. The whole approach exhibits novel, data-driven, algorithmic steps that can be summarized as follows:

• The construction of a IDFCG that incorporates dominant types of interactions, either intra- (e.g., 𝜃-𝜃) or inter-frequency [phase-to-amplitude coupling (PAC) (e.g., 𝜃-γ)] coupling.
• The application of a new thresholding scheme termed OMSTs as a topological filtering applied to DFCG to extract a “true” network topology.
• The VQ modeling of network metric time series (NMTS) based on nodal Laplacian eigenvalues for prototyping the spatiotemporal dynamics of both control and MCI subjects.
• Modeling of the switching behavior of brain states as a Markovian chain
• The validation of the whole approach to a second blind dataset achieving an 85% classification accuracy for the CENT ROI representation scheme compared to 70% for the PCA scheme
• Regions of interest representation scheme matters on the designing of connectomic biomarker in general and also for MCI
• Canonical correlation analysis between chronnectomics and MMSE revealed that the DT of brain states associates strongly with the CCA mode of MMSE variability.

We proved that the VQ modeling of NMTS^eigen^ is an effective approach to extract an overcomplete dictionary for the representation of DFC that can accurately classify subjects as either control or MCI based on their resting state MEG activity. Adopting a static network analysis, the classification accuracy was 12 out of 20, demonstrating the need of a DFC approach for studying resting brain dynamics (Dimitriadis et al., 2010b, 2012a,c, 2013a,b, 2015a,b, 2016a, 2017a,b, 2018a,b; Allen et al., 2014; Damaraju et al., 2014; Kopell et al., 2014).

The capture of time-varying coupling between variables is a topic that has been heavily studied in other fields and in communications for signal processing in particular. However, the specific application to whole-brain functional connectivity is relatively new (Sakoglu et al., 2010; Dimitriadis et al., 2013a; Calhoun et al., 2014), and its application to brain-imaging data poses particular challenges, which are the topic of active current research. One important challenge is how to best identify relevant features from the high-dimensional brain imaging data. The main algorithms used for manipulating functional brain network dynamics in fMRI are group ICA (Calhoun and Adali, 2012) or spatial-constrained ICA (Lin et al., 2013) and tensor decompositions (Acar and Yener, 2009). To characterize the dynamics of time-varying connectivity brain patterns, the basic approach is the metastate analysis based on the sliding window or more adaptive approach (Dimitriadis et al., 2013a, 2015d; Damaraju et al., 2014; Nomi et al., 2016). From the dynamic connectivity patterns, FCμstates are extracted that are “quasi-stable” distinct brain states. Then, the state vectors can be modeled via a Markovian chain (Dimitriadis et al., 2013a, 2015b; Calhoun et al., 2014; Damaraju et al., 2014).

Cross-frequency coupling mechanisms support the brain interactions across space over multiple temporal scales (Canolty and Knight, 2010; Fell and Axmacher, 2011). Computational models have explored the theoretical advantages of the existence of cross frequency coupling (Lisman and Idiart, 1995; Neymotin et al., 2011). These models untangled the major mechanisms of the importance of CFC, which may serve as the brain’s neural syntax. Segmentation of spike trains into cell clusters (“letters”) and sequences of assemblies (neural “words”) are supported by the existing syntactic rules (Buzsaki, 2010).

In the present study, we demonstrated a methodology whose main scope is to provide a framework for modeling DFCG into a repertoire of distinct “quasi-static” brain states called FCμstates. Here, we modeled the NMTS derived from the DFC patterns expressed via the Laplacian eigenvalues (Dimitriadis et al., 2015d, 2017b, 2018b). After extracting the virtual source time series, we followed an algorithmic approach with the main aim of minimizing the effect of *a priori* selection of variables that can minimize the reproducibility of the results. The main steps of the proposed methodology are: (1) the construction of one integrated DFC per subject—which incorporates the DICM per each pair of brain areas and at every temporal segment, (2) the application of a data-driven topological filtering scheme to reveal the backbone of the network topology at every temporal segment, (3) the estimation of Laplacian eigenvalues to extract the so-called NMTSeigen (Dimitriadis et al., 2010a, 2015d, 2017b, 2018b), (4) the modeling of these NMTS^eigen^ via a vector-quantization approach, and (5) the validation of the whole approach to a second blind dataset.

The analysis of the spatiotemporal evolution of Laplacian eigenvalues during the training phase revealed three prototypical brain states (FCμstates). For a better illustration of the FCGs linked to the prototypical eigenvalues, we assigned the 90 AAL brain areas to five well-known brain networks. In Figure 4, we mapped the average functional strength between ROIs belonging to every pair of brain networks while the size and color of every node define the within-brain network degree. The most connected brain networks in FCμstates are the DMN and CO. CO plays a key role in working memory mechanisms (Wallis et al., 2015) while cognitive complaints related to AD are linked to alterations of resting-state brain networks and mostly FPN and DMN (Contreras et al., 2017). The functional coupling strength between FPN and DMN was significantly higher for HC compared to MCI for FCμstates 1 and 3 (Figure 4). The functional strength between FPN-DMN was positively correlated with a better episodic memory performance (Contreras et al., 2017).

Well-known and novel chronnectomics were estimated from the Markovian (symbolic) Chain that describes the evolution of brain states. We detected significantly higher flexibility and complexity for HC as compared to MCI described from FI and CI, correspondingly (Figure 7). A summarization derived from OT and DT revealed a significant trend: HC subjects spent significantly more time compared to MCI in FCμstates 1 and 3 while MCI spent significantly more time in the second FCμstate (Figure 8). Following a CCA analysis between the extracted chronnectomics and the MMSE score, we found a significant contribution of the DT for the three NMTS^eigen^. OC related to the 2nd NMTS^eigen^ did not associate with the CCA mode of MMSE variability (Figure 11).

In the era of data sharing and aggregating large datasets from different research groups worldwide who contribute to large consortiums, it is important to test the reproducibility of the proposed biomarkers (Abraham et al., 2017). Our study is a first step in this direction to diminish the effect of any arbitrary selection of algorithmic steps up to the extraction of biomarkers. The next step is to extend the analysis in larger populations from different sites and MEG scanners. A recent study showed that 70 percent of the preclinical research from academic labs could not be replicated (Collins and Tabak, 2014). Abraham’s work is one of the very first neuroimaging studies that lays the ground for the reliability and reproducibility of biomarkers extracted from neuroimaging data.

There is a large body of research based on different imaging methods covering various temporal and spatial scales that documents the association of electrophysiological rhythms with distinct cognitive processes within narrowly or broadly anatomical areas (for review, see Engel et al., 2001; Buszaky, 2006; Siegel et al., 2012; Başar and Güntekin, 2013). For example, low-frequency δ rhythms (1–4 Hz) are known to coordinate large portions of the brain (Fujisawa and Buzsaki, 2011; Nacher et al., 2013) while γ oscillations play a dominant role in stimulus processing and detection is shown to be locally anatomically constrained (Engel et al., 2001). Recently, an extension of Brodmann’s areas was suggested in order to associate distinct anatomical areas with preferable connectivity estimators and cognitive functions in both normal and brain disease/disorder populations as an initial step toward summarizing the large body of current brain connectivity research (Başar and Düzgün, 2016).

In the last few years, an increasing number of studies appeared studying CFC at resting state (Antonakakis et al., 2016a), during cognitive tasks (Dimitriadis et al., 2015a,c, 2016a,b) and in various brain diseases and disorders such as mild traumatic brain injury (Antonakakis et al., 2016a), amnestic MCI (Dimitriadis et al., 2015a), dyslexia (Dimitriadis et al., 2016a), schizophrenia (Kirihara et al., 2012), etc. It has been suggested that CFC is the key mechanism for the integration of information between anatomical distribution subsystems that function on a dominant frequency (Canolty and Knight, 2010; Jirsa and Muller, 2013; Florin and Baillet, 2015). However, only a few MEG studies have explored CFC at resting state (Antonakakis et al., 2015, 2016a,b; Florin and Baillet, 2015) and especially in a more dynamic fashion (Dimitriadis et al., 2015a, 2016a; Antonakakis et al., 2016b).

MEG source connectivity is at a mature level compared to a decade ago (Ioannides et al., 2012), and it is an active research area aimed at improving many aspects of “true” brain connectivity (Schoffelen and Gross, 2009; Colclough et al., 2016). The most significant issue is the parcellation of the cerebral cortex. In many cases, the AAL template (90 ROIs) is feasible for the detection of those changes induced by a specific task or obtained after comparing different groups. But in others such as the design of a reliable connectomic biomarker, there is a need to oversample more than 90 areas. The FC, which is directly linked to functional parcellation of the cerebral cortex, is an active area, which will further improve both the interpretation and the predictive power of source connectivity of many brain diseases such as MCI. The solution of a functional parcellation template for MEG source connectivity will improve the classification performance on the source level with the additional advantage, compared to sensor level, of facilitating the anatomical interpretation of the results.

Adopting the same framework and including also stable and progressive MCI groups, we will attempt to connect DCB with neuropsychological measures and cognitive scores (Cuesta et al., 2014, 2015). It is evident that a multifactorial model that includes cognition, neuropsychological measures and anatomical information can reliably predict the conversion from MCI to DAT, while genetic variation of risk genes like the APOE-e4 allele or cognitive reserve might play a secondary role (López et al., 2016).

Going one step further from our previous studies demonstrating the significance of a DCB (Dimitriadis et al., 2013b, 2015b), where we used network microstates extracted from DFCG patterns, in the present study we introduced a modeling approach of NMTS^eigen^ estimated over DFCGs that preserve the dominant type of coupling (intra- or inter-frequency intrinsic coupling mode). Our study demonstrates the effectiveness of the data-driven analytic pipeline tailored to DFCG to the correct classification of a blind dataset based on control and MCI subjects compared to a static connectivity approach. Given these outcomes, the need is evident over the next years to adopt data-driven techniques that will not introduce bias, subjectivity and assumptions in neuroimaging datasets and also to improve the reproducibility of the outcome in large databases.

In magnetoencephalography (MEG) the conventional approach to source reconstruction is to solve the underdetermined inverse problem independently over time and space. Different algorithms have been proposed so far with alternative regularization procedures of space and time as with a Gaussian random field model (Solin et al., 2016).

Commonly used techniques include the minimum-norm estimate (MNE) (Hämäläinen and Ilmoniemi, 1994) and Linearly Constrained Minimum-Variance (LCMV) beamformer (Van Veen et al., 1997). It is in the right direction to compare the consistency of the outcome of the current study with alternative inverse solution algorithms to measure their consistency and sensitivity to the design of connectomic biomarkers tailored to MCI.

## Conclusion

In this study, we presented a novel DCM for the prediction of MCI from an age-matched control group validated over a blind dataset. The novelties of the proposed analytic scheme are the incorporation in the DFCGs of the DICM (DICM, either intra- or inter-frequency coupling based on PAC), the adaptation of a novel data-driven thresholding scheme based on OMSTs, the estimation of Laplacian eigenvalues across time and the extraction of prototypical network microstates (FCμstates) for both the control and MCI group.

It is important for the near future to work in source space on MCI subjects that convert to AD after a following up study to further validate the proposed scheme as a potential tool of clinical importance. It would also be interesting to explore how the Apoe-e4 allele can induce changes to the DFC of spontaneous activity. Moreover, multimodal neuroimaging biomarkers is a novel trend that will further be validated (Jack et al., 2016).

## Data Availability

All datasets generated for this study are included in the manuscript and/or the Supplementary Files.

## Author Contributions

SD conceptualized the research analysis, methods, and design, data analysis, and drafting the manuscript. ML acquired the data. ML, FM, and EP criticized the revision of the manuscript. All authors read and approved the final version of the manuscript.

## Conflict of Interest Statement

The authors declare that the research was conducted in the absence of any commercial or financial relationships that could be construed as a potential conflict of interest.
